# Comparative Clinical Outcomes of Laser-Assisted Periodontal Therapy Versus Conventional Scaling and Root Planing in Patients With Chronic Periodontitis: A Prospective Nonrandomized Study

**DOI:** 10.7759/cureus.114079

**Published:** 2026-08-06

**Authors:** Chandrashekar Buregoni, Neelam Das, Ilakiya Mathi, Lakshminarayanan S, Jawahar Raman L, Manoj Margabandhu, Sajidali S Saiyad

**Affiliations:** 1 Department of Periodontology, Tirumala Institute of Dental Sciences and Research Center, Hyderabad, IND; 2 Department of Periodontology, Sri Sai College of Dental Surgery, Hyderabad, IND; 3 Department of Periodontology, Adhiparasakthi Dental College and Hospital, Melmaruvathur, IND; 4 Department of Periodontics, Mahatma Gandhi Postgraduate Institute of Dental Sciences, Pondicherry, IND; 5 Department of Periodontology, Mahatma Gandhi Postgraduate Institute of Dental Sciences, Pondicherry, IND; 6 Department of Physiology, Pacific Medical College and Hospital, Pacific Medical University, Udaipur, IND

**Keywords:** chronic periodontitis, clinical attachment gain, clinical attachment level, diode laser, laser-assisted periodontal therapy, non-surgical periodontal therapy, periodontal treatment outcomes, probing pocket depth, scaling and root planing

## Abstract

Background

Scaling and root planing (SRP) is the standard nonsurgical treatment for chronic periodontitis; however, complete mechanical debridement may be limited in deep periodontal pockets, prompting interest in adjunctive laser therapy to enhance clinical outcomes.

Objective

To compare the clinical effectiveness and safety of adjunctive 940-nm diode laser therapy combined with SRP versus SRP alone in patients with chronic periodontitis.

Methods

This prospective nonrandomized comparative clinical study included 60 patients with Stage II-III, Grade B periodontitis who were allocated to receive either SRP alone (n = 30) or SRP with adjunctive 940-nm diode laser therapy (n = 30) based on participant preference following an informed discussion at a single tertiary care center. Clinical attachment level (CAL) was the primary outcome. Secondary outcomes included probing pocket depth (PPD), plaque index (PI), gingival index (GI), bleeding on probing (BOP), and treatment-related safety. Clinical assessments were performed at baseline and at 4, 12, and 24 weeks by a blinded, calibrated examiner who was not involved in treatment delivery. Within-group comparisons were analyzed using paired t-tests, between-group comparisons using independent t-tests, and longitudinal changes using repeated-measures ANOVA. Multivariable linear regression was used to identify independent predictors of CAL gain.

Results

Both treatment groups demonstrated significant improvements in all clinical parameters over 24 weeks (p < 0.001). Compared with SRP alone, adjunctive diode laser therapy produced significantly greater PPD reduction (3.58 ± 0.64 vs. 3.22 ± 0.58 mm; p = 0.026) and CAL gain (3.42 ± 0.78 vs. 3.01 ± 0.69 mm; p = 0.034) at 24 weeks. Significant time × treatment interactions were observed for PPD and CAL (p < 0.05). Multivariable regression confirmed adjunctive laser therapy as an independent predictor of CAL gain. No significant between-group differences were observed in PI, GI, BOP, or adverse events.

Conclusions

Adjunctive 940-nm diode laser therapy was associated with additional clinical benefits over conventional SRP, including enhanced periodontal pocket reduction and attachment gain, without compromising safety. These findings support its use as a complementary nonsurgical treatment modality for appropriately selected patients with chronic periodontitis and provide prospective clinical evidence to guide periodontal practice.

## Introduction

Periodontitis is a biofilm-associated inflammatory disease characterized by a dysregulated host immune response that results in the progressive destruction of periodontal supporting tissues, leading to periodontal pocket formation, clinical attachment loss (CAL), and alveolar bone resorption. If left untreated, it may ultimately result in tooth loss. Beyond its oral manifestations, periodontitis has been associated with systemic conditions such as diabetes mellitus and cardiovascular disease, highlighting the importance of effective periodontal management as part of comprehensive patient care [1-3]. With a global prevalence exceeding 40%, periodontitis represents a substantial public health burden [2].

Scaling and root planing (SRP) remains the cornerstone of nonsurgical periodontal therapy, achieving its effects through the disruption of subgingival biofilm and the removal of calculus deposits. However, complete mechanical debridement may be challenging in deep periodontal pockets, furcation areas, and anatomically complex root surfaces, where residual microorganisms may persist and compromise periodontal healing [4,5].

Adjunctive therapies have therefore been investigated to enhance the effectiveness of SRP. Among these, 940-nm diode laser therapy has gained increasing attention because of its antimicrobial, hemostatic, and photobiomodulatory properties, which may improve periodontal wound healing in addition to reducing the subgingival microbial burden [6,7]. Recent systematic reviews and meta-analyses suggest that adjunctive diode laser therapy may provide additional improvements in probing pocket depth (PPD) reduction and CAL gain; however, the magnitude and consistency of these benefits remain uncertain because of substantial heterogeneity in laser parameters, treatment protocols, disease severity, and follow-up duration across published studies [6,8-10].

Although several studies have evaluated adjunctive diode laser therapy, prospective comparative studies using standardized treatment protocols and clinically relevant follow-up periods remain limited, particularly among Indian populations. This study addresses this gap by evaluating a standardized 940-nm diode laser protocol under routine clinical practice conditions, with blinded outcome assessment, a 24-week longitudinal follow-up, and multivariable regression analysis, thereby providing region-specific evidence to support evidence-based periodontal practice.

We hypothesized that adjunctive 940-nm diode laser therapy combined with conventional SRP would be associated with greater CAL gain and PPD reduction than SRP alone over a 24-week follow-up period. The primary objective was to compare the change in CAL between the treatment groups from baseline to 24 weeks. The secondary objectives were to compare changes in PPD, plaque index (PI), gingival index (GI), bleeding on probing (BOP), and treatment-related safety outcomes.

## Materials and methods

Study design and rationale

The present investigation employed a prospective, nonrandomized comparative clinical design to examine the effectiveness of DL-assisted periodontal treatment as an adjunct to conventional SRP versus SRP alone in patients with periodontitis.

This study was approved by the Institutional Ethics Committee (IEC) of Pacific Medical College and Hospital, Udaipur, Rajasthan, India (Approval No.: PMU/PMCH/IEC/GEN/2023/154; approval date: February 13, 2023). Written informed consent was obtained from all participants before enrollment. All study procedures were conducted in accordance with the ethical principles of the Declaration of Helsinki (2013 revision) and applicable institutional guidelines.

Study population and sample size calculation

This prospective, nonrandomized comparative clinical study was conducted in the Department of Periodontology, Pacific Medical College and Hospital, Pacific Medical University, Udaipur, Rajasthan, India, between March 2023 and December 2024. Periodontitis was diagnosed according to the 2017 World Workshop Classification of Periodontal and Peri-Implant Diseases and Conditions [11]. Participants were enrolled according to the predefined inclusion and exclusion criteria shown in Table 1.

**Table 1:** Inclusion and exclusion criteria for participant enrollment. Eligibility was determined before treatment allocation. Periodontitis was diagnosed according to the 2017 World Workshop on the Classification of Periodontal and Peri-Implant Diseases and Conditions. Participants were enrolled only if all inclusion criteria were met and none of the exclusion criteria applied. PPD: Probing pocket depth; CAL: Clinical attachment level; BOP: Bleeding on probing.

Inclusion Criteria	Exclusion Criteria
Systemically healthy adults aged 30-65 years	Periodontal therapy within the previous 12 months
Diagnosis of Stage II-III, Grade B periodontitis according to the 2017 World Workshop on the Classification of Periodontal and Peri-Implant Diseases and Conditions	Antibiotic therapy within the preceding 6 months
Presence of ≥20 natural teeth	Systemic diseases or conditions known to affect periodontal healing (e.g., immunodeficiency or malignancy)
At least two noncontiguous sites with PPD ≥5 mm and CAL ≥3 mm	Pregnancy or lactation
BOP at the selected sites	Heavy smoking (>10 cigarettes/day)
Radiographic evidence of alveolar bone loss	Teeth with advanced furcation involvement or deemed nonrestorable

The sample size was calculated using G*Power version 3.1.9.7 (Heinrich Heine University, Düsseldorf, Germany) for the comparison of two independent groups with equal allocation. Based on the primary outcome (change in CAL), a clinically meaningful between-group difference of 1.0 mm, a common SD of 1.2 mm, a two-sided α of 0.05, and 80% power, the minimum required sample size was 23 participants per group. After adding 20% for anticipated dropout, the target sample size increased to 28 participants per group. To ensure adequate statistical power, 30 participants were enrolled in each group, yielding a total sample of 60 participants [2,12].

Treatment allocation

Eligible participants were allocated to either the SRP plus DL group or the SRP alone group according to their treatment preference after receiving standardized verbal and written information regarding the available treatment options, including the procedures, potential benefits, risks, expected outcomes, and costs, where applicable. Written informed consent was obtained before treatment allocation.

As treatment allocation was based on participant preference following an informed discussion with the treating periodontist, randomization and allocation concealment were not performed. To minimize measurement bias, all clinical outcome assessments were conducted by a single trained and calibrated examiner who remained blinded to treatment allocation throughout the study. The treating periodontist was not involved in the outcome assessment or statistical analysis.

Interventions

All participants received standardized oral hygiene instructions, including training in the modified Bass brushing technique using a soft-bristled toothbrush. During the preparatory phase, full-mouth supragingival scaling and polishing were performed using an ultrasonic scaler (Cavitron® Select SPS, Dentsply Sirona, Charlotte, NC, USA). One week later, baseline clinical measurements were recorded, and treatment was performed under local anesthesia using 2% lidocaine with 1:100,000 epinephrine.

In both groups, SRP was performed using ultrasonic inserts and Gracey curettes (Hu-Friedy®, Chicago, IL, USA) until all supragingival and subgingival deposits had been removed and a smooth root surface had been achieved. In the SRP + DL group, adjunctive irradiation was performed using a 940-nm DL (Biolase® Epic X, Biolase Inc., Irvine, CA, USA) operated in continuous-wave mode at 1.0 W, with an energy density of approximately 4.5 J/cm². A 300-μm initiated fiber tip was introduced to the base of the periodontal pocket and moved coronally using a sweeping motion for 20 seconds per site. Each site received a single irradiation cycle, with the fiber maintained parallel to the root surface.

Following treatment, participants were instructed to avoid mechanical plaque control at the treated sites for 48 hours and to rinse with 0.12% chlorhexidine gluconate twice daily for 14 days, after which routine oral hygiene was resumed. Supportive periodontal maintenance, including reinforcement of oral hygiene instructions and supragingival scaling when indicated, was provided at each follow-up visit. No protocol deviations occurred during the study.

Clinical periodontal examination

Clinical periodontal examinations were performed at baseline and at 4, 12, and 24 weeks after treatment by a single trained and calibrated examiner who remained blinded to treatment allocation. Measurements were obtained using a Williams periodontal probe (Hu-Friedy®, Chicago, IL, USA) with a standardized probing force. To enhance measurement reproducibility, a customized autopolymerizing acrylic occlusal stent with vertical guiding grooves was fabricated for each participant to standardize probe angulation and measurement sites throughout the study.

The primary outcome was the change in CAL from baseline to 24 weeks. Secondary outcomes included PPD, PI, GI, and BOP. Clinical measurements were recorded at six sites per tooth: mesiobuccal, midbuccal, distobuccal, mesiolingual, midlingual, and distolingual. PI and GI were recorded using the Silness and Löe Plaque Index and the Löe and Silness Gingival Index, respectively. PPD was measured as the distance from the gingival margin to the base of the periodontal pocket, whereas CAL was measured from the cementoenamel junction, or the restoration margin where applicable, to the base of the pocket. BOP was recorded as present or absent based on whether bleeding occurred within 15 seconds of gentle probing. All linear measurements were recorded to the nearest millimeter [1,11,13-15].

Examiner calibration

Before the commencement of the study, the examiner underwent calibration to ensure the reproducibility of periodontal measurements. The reproducibility of continuous periodontal variables (PPD and CAL) was evaluated using the intraclass correlation coefficient (ICC), whereas agreement for the dichotomous BOP recordings was assessed using Cohen’s kappa statistic. The obtained ICC values were 0.95 for PPD and 0.93 for CAL, and Cohen’s κ was 0.89 for BOP, indicating excellent intraexaminer reliability.

Ethical considerations regarding clinical indices

Permission to use the PI, GI, PPD, CAL, and BOP assessment methods was not required because these are internationally accepted, standardized periodontal assessment tools published in the scientific literature and freely available for use in clinical research.

Statistical analysis

Statistical analyses were performed using R version 4.6.1 (R Foundation for Statistical Computing, Vienna, Austria), and statistical figures were generated using the ggplot2 package. The study flow diagram and schematic illustrations were created using diagrams.net (draw.io). Continuous variables are presented as means ± SDs, and categorical variables as frequencies and percentages. Data normality was assessed using the Shapiro-Wilk test. Between-group and within-group comparisons were performed using parametric tests (independent or paired t-tests, respectively) or nonparametric tests (Mann-Whitney U or Wilcoxon signed-rank tests, respectively), as appropriate. Categorical variables were compared using the chi-square test or Fisher’s exact test. Effect sizes (Cohen’s d, phi coefficient, Cramér’s V, and partial η²) were calculated where appropriate.

The effects of time, treatment, and the time × treatment interaction were evaluated using repeated-measures analysis of variance (RM-ANOVA), with the Greenhouse-Geisser correction applied when required (ε: PPD = 0.72; CAL = 0.68). Multivariable linear regression was performed to identify independent predictors of CAL gain, adjusting for baseline CAL, baseline PPD, age, sex, smoking status, and diabetes mellitus. Model assumptions were verified by assessing residual normality, homoscedasticity, multicollinearity (variance inflation factor < 2.0), and influential observations (Cook’s distance and leverage values). All tests were two-tailed, with statistical significance defined as p < 0.05. The Bonferroni correction was applied for multiple comparisons where appropriate. As no data were missing, a complete-case analysis was performed.

## Results

Baseline characteristics

**Figure 1:**
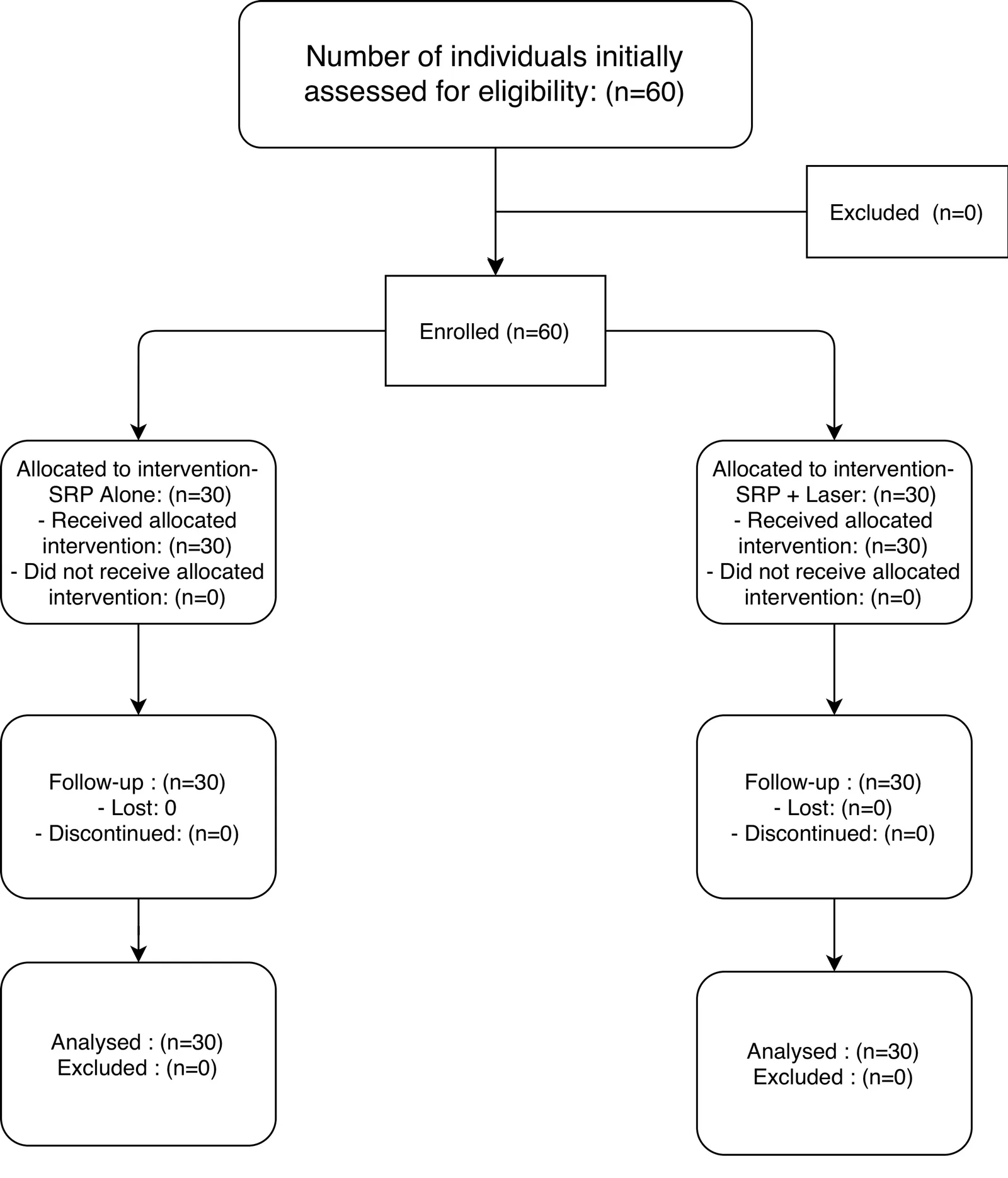
Study flow. Participant flow diagram showing the enrollment, allocation, follow-up, and analysis of study participants. A total of 60 participants (30 per group) with Stage II-III, Grade B periodontitis were enrolled in this prospective nonrandomized comparative clinical study. All participants completed the 24-week follow-up period, with no withdrawals or losses to follow-up.

Sixty participants with periodontitis were enrolled, with 30 participants allocated to the SRP alone group and 30 participants allocated to the SRP plus DL group based on participant preference. All participants completed the 24-week follow-up without withdrawal, resulting in no missing outcome data (Figure 1).

Baseline demographic and clinical characteristics are summarized in Table 2. No statistically significant between-group differences were observed in age, sex distribution, smoking status, systemic health status, tooth distribution, defect morphology, or baseline periodontal parameters, including PI, GI, PPD, CAL, BOP, and the number of sites with PPD ≥5 mm (all p > 0.05).

**Table 2:** Baseline demographic and clinical characteristics of study participants. SRP: Scaling and root planing; PI: Plaque index; GI: Gingival index; PPD: Probing pocket depth; CAL: Clinical attachment level; BOP: Bleeding on probing; df: Degrees of freedom; d: Cohen’s d; φ: Phi coefficient. Statistical analyses: Independent-samples t-test for continuous variables and chi-square test for categorical variables. Effect sizes were interpreted as follows: Cohen’s d (small = 0.2, medium = 0.5, and large = 0.8); phi coefficient (small = 0.1, medium = 0.3, and large = 0.5); and Cramér’s V (small = 0.1, medium = 0.3, and large = 0.5). Note: No statistically significant differences were observed between the groups at baseline for any demographic or clinical parameter (p > 0.05 for all comparisons), confirming baseline comparability between the treatment groups. The degree of balance observed was attributable, in part, to the alternating allocation method used, which systematically minimized between-group differences during enrollment.

Characteristic	SRP-Alone Group (n = 30)	SRP + Diode Laser Group (n = 30)	Test Statistic	df	p-value	Effect Size
Age (years), mean ± SD	44.53 ± 9.87	46.20 ± 10.34	t = -0.642	58	0.523	d = 0.17
Age range (years)	28-62	30-65	-	-	-	-
Sex, n (%)						
Male	17 (56.7%)	16 (53.3%)	χ² = 0.067	1	0.796	φ = 0.033
Female	13 (43.3%)	14 (46.7%)				
Smoking status, n (%)						
Nonsmoker	22 (73.3%)	23 (76.7%)	χ² = 0.073	1	0.787	φ = 0.035
Smoker (≤10 cigarettes/day)	8 (26.7%)	7 (23.3%)				
Systemic conditions, n (%)						
Type 2 diabetes mellitus	4 (13.3%)	4 (13.3%)	χ² = 0.000	1	1	φ = 0.000
None	26 (86.7%)	26 (86.7%)				
Baseline clinical parameters, mean ± SD						
Plaque index (PI)	1.82 ± 0.31	1.79 ± 0.28	t = 0.394	58	0.695	d = 0.10
Gingival index (GI)	1.56 ± 0.42	1.61 ± 0.38	t = -0.486	58	0.629	d = 0.13
Probing pocket depth (PPD), mm	5.23 ± 0.76	5.31 ± 0.82	t = -0.395	58	0.695	d = 0.10
Clinical attachment level (CAL), mm	4.68 ± 0.89	4.75 ± 0.93	t = -0.299	58	0.766	d = 0.08
Bleeding on probing (BOP), %	68.40 ± 18.92	71.20 ± 19.45	t = -0.565	58	0.574	d = 0.15
Number of sites with PPD ≥5 mm, mean ± SD	6.43 ± 2.87	6.97 ± 3.12	t = -0.702	58	0.485	d = 0.18
Tooth type distribution, n (%)						
Incisors/canines	32 (35.6%)	29 (32.2%)	χ² = 1.248	2	0.536	Cramér’s V = 0.144
Premolars	28 (31.1%)	34 (37.8%)				
Molars	30 (33.3%)	27 (30.0%)				
Defect morphology, n (%)						
Horizontal	46 (51.1%)	41 (45.6%)	χ² = 0.872	2	0.647	Cramér’s V = 0.120
Vertical (infrabony)	27 (30.0%)	33 (36.7%)				
Combined	17 (18.9%)	16 (17.8%)				

Longitudinal changes in clinical outcomes

Changes in periodontal parameters over the 24-week follow-up period are presented in Table 3. Both treatment groups demonstrated significant improvements from baseline in all evaluated clinical parameters, including PI, GI, PPD, CAL, and BOP, at every follow-up visit (all within-group p < 0.001). The largest improvements occurred during the first four weeks following treatment, whereas more gradual changes were observed thereafter.

**Table 3:** Longitudinal clinical outcomes at baseline and at 4, 12, and 24 weeks. SRP: Scaling and root planing; PI: Plaque index; GI: Gingival index; PPD: Probing pocket depth; CAL: Clinical attachment level; BOP: Bleeding on probing. † Within-group change from baseline, assessed using a paired t-test. *p < 0.001 for all within-group changes. ‡ Between-group difference (SRP + laser minus SRP alone) at each time point, assessed using an independent-samples t-test. § p-value for the between-group comparison at each time point. †† Statistically significant at p < 0.05. Note: Both treatment groups demonstrated statistically significant improvements in all periodontal parameters at all follow-up time points (p < 0.001 for all within-group comparisons). The SRP + laser group showed a significantly greater reduction in PPD at 24 weeks (p = 0.026) and greater CAL gain at 24 weeks (p = 0.034) than the SRP-alone group. Differences in PI, GI, and BOP were not statistically significant at any time point, including the difference in BOP at 24 weeks (p = 0.084).

Parameter	Time Point	SRP-Alone Group (n = 30), Mean ± SD	SRP + Laser Group (n = 30), Mean ± SD	Within-Group Change From Baseline (95% CI)†	Between-Group Difference (95% CI)‡	P-value§
Plaque index (PI)	Baseline	1.82 ± 0.31	1.79 ± 0.28	-	0.03 (-0.12, 0.18)	0.695
	4 weeks	0.92 ± 0.27	0.86 ± 0.24	-0.90 (-1.02, -0.78)*	0.06 (-0.07, 0.19)	0.361
	12 weeks	0.74 ± 0.25	0.67 ± 0.22	-1.08 (-1.20, -0.96)*	0.07 (-0.05, 0.19)	0.249
	24 weeks	0.58 ± 0.24	0.51 ± 0.21	-1.24 (-1.38, -1.10)*	0.07 (-0.04, 0.18)	0.233
Gingival index (GI)	Baseline	1.56 ± 0.42	1.61 ± 0.38	-	-0.05 (-0.25, 0.15)	0.629
	4 weeks	0.73 ± 0.28	0.64 ± 0.25	-0.83 (-0.96, -0.70)*	0.09 (-0.05, 0.23)	0.193
	12 weeks	0.56 ± 0.22	0.48 ± 0.20	-1.00 (-1.14, -0.86)*	0.08 (-0.03, 0.19)	0.151
	24 weeks	0.42 ± 0.19	0.35 ± 0.16	-1.14 (-1.30, -0.98)*	0.07 (-0.02, 0.16)	0.127
Probing pocket depth (PPD), mm	Baseline	5.23 ± 0.76	5.31 ± 0.82	-	-0.08 (-0.49, 0.33)	0.695
	4 weeks	4.26 ± 0.72	4.08 ± 0.74	-0.97 (-1.22, -0.72)*	0.18 (-0.19, 0.55)	0.339
	12 weeks	3.87 ± 0.68	3.54 ± 0.66	-1.36 (-1.62, -1.10)*	0.33 (-0.02, 0.68)	0.063
	24 weeks	3.58 ± 0.64	3.22 ± 0.58	-1.65 (-1.92, -1.38)*	0.36 (0.05, 0.67)	0.026††
Clinical attachment level (CAL), mm	Baseline	4.68 ± 0.89	4.75 ± 0.93	-	-0.07 (-0.54, 0.40)	0.766
	4 weeks	4.12 ± 0.84	4.02 ± 0.86	-0.56 (-0.78, -0.34)*	0.10 (-0.34, 0.54)	0.649
	12 weeks	3.76 ± 0.82	3.48 ± 0.78	-0.92 (-1.18, -0.66)*	0.28 (-0.14, 0.70)	0.183
	24 weeks	3.42 ± 0.78	3.01 ± 0.69	-1.26 (-1.58, -0.94)*	0.41 (0.04, 0.78)	0.034††
Bleeding on probing (BOP), %	Baseline	68.40 ± 18.92	71.20 ± 19.45	-	-2.80 (-12.52, 6.92)	0.574
	4 weeks	44.20 ± 16.84	38.80 ± 17.21	-24.20 (-30.56, -17.84)*	5.40 (-3.22, 14.02)	0.221
	12 weeks	34.80 ± 15.62	29.60 ± 15.84	-33.60 (-40.18, -27.02)*	5.20 (-2.82, 13.22)	0.21
	24 weeks	28.60 ± 14.32	22.40 ± 12.87	-39.80 (-46.23, -33.37)*	6.20 (-0.78, 13.18)	0.084

Between-group comparisons showed no statistically significant differences at baseline or at 4 or 12 weeks for any periodontal parameter. By 24 weeks, however, participants receiving adjunctive DL therapy exhibited significantly greater reductions in PPD (mean difference, 0.36 mm; 95% CI, 0.05-0.67; p = 0.026) and significantly greater CAL gain (mean difference, 0.41 mm; 95% CI, 0.04-0.78; p = 0.034) than those in the SRP-alone group. No statistically significant between-group differences were observed in PI, GI, or BOP at any follow-up assessment. The difference in BOP at 24 weeks was also not statistically significant (p = 0.084).

**Figure 2:**
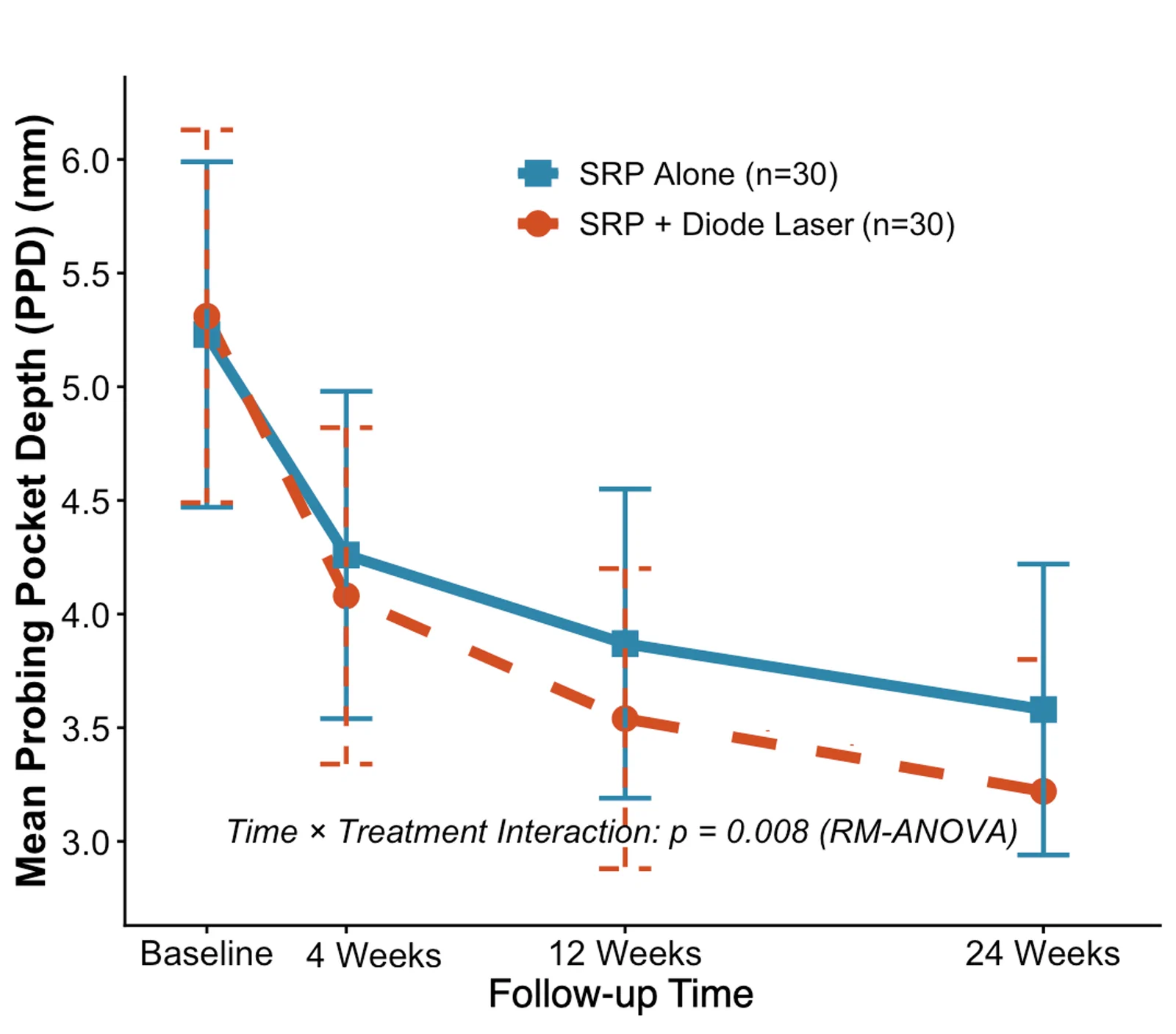
Changes in mean PPD over 24 weeks. Changes in mean PPD over 24 weeks in the SRP-alone (n = 30) and SRP + diode laser (n = 30) groups. Data are presented as means ± SDs, with error bars representing SDs. Both groups demonstrated significant reductions in PPD over time (p < 0.001 for all within-group comparisons). The SRP + diode laser group showed a significantly greater reduction at 24 weeks than the SRP-alone group (*p = 0.026; independent-samples t-test). The time × treatment interaction was also significant (p = 0.008; repeated-measures analysis of variance with the Greenhouse-Geisser correction). SRP: Scaling and root planing; PPD: Probing pocket depth.

Both treatment groups showed significant reductions in PPD over 24 weeks, with the SRP + laser group demonstrating a greater reduction at the final time point (Figure 2).

**Figure 3:**
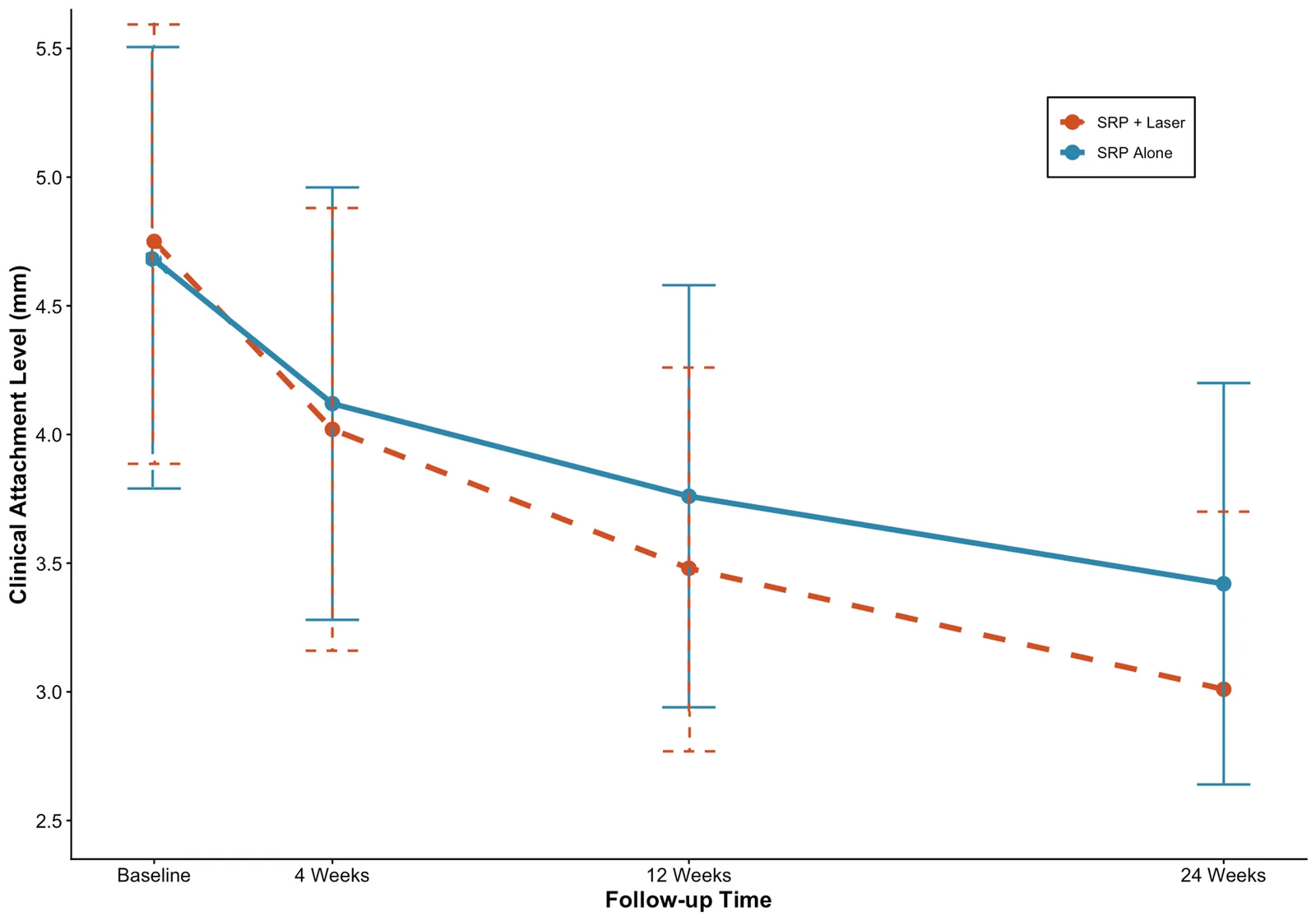
Changes in CAL over 24 weeks. Changes in mean CAL over 24 weeks in the SRP-alone (n = 30) and SRP + diode laser (n = 30) groups. Data are presented as means ± SDs, with error bars representing SDs. Both groups demonstrated significant improvements in CAL over time (p < 0.001 for all within-group comparisons, except at four weeks in the SRP + diode laser group (p = 0.002)). The SRP + diode laser group showed significantly greater CAL gain at 24 weeks than the SRP-alone group (*p = 0.034; independent-samples t-test). The time × treatment interaction was also significant (p = 0.022; repeated-measures analysis of variance with the Greenhouse-Geisser correction). SRP: Scaling and root planing; CAL: Clinical attachment level.

CAL improved significantly in both groups throughout the study, with the SRP + laser group exhibiting greater CAL gain at 24 weeks (Figure 3).

Repeated-measures analysis

Repeated-measures ANOVA demonstrated significant main effects of time for all periodontal parameters, consistent with improvement throughout the study irrespective of treatment allocation (all p < 0.001). Significant treatment effects were observed for PPD (p = 0.004) and CAL (p = 0.016), indicating overall between-group differences favoring the SRP plus DL group.

Significant time × treatment interactions were also identified for PPD (F = 4.05, p = 0.008, partial η² = 0.065) and CAL (F = 3.30, p = 0.022, partial η² = 0.054), indicating that the magnitude of improvement over time differed between the treatment groups. No significant interaction effects were observed for PI, GI, or BOP (all p > 0.05). The results are summarized in Table 4.

**Table 4:** Repeated-measures analysis of variance (RM-ANOVA) for clinical parameters. df: Degrees of freedom; SRP: Scaling and root planing; PI: Plaque index; GI: Gingival index; PPD: Probing pocket depth; CAL: Clinical attachment level; BOP: Bleeding on probing. Partial η² (partial eta-squared) measures effect size: small = 0.01, medium = 0.06, and large = 0.14. Statistical analysis: Repeated-measures ANOVA with the Greenhouse-Geisser correction applied for violations of sphericity. The factors were time (four levels: baseline and 4, 12, and 24 weeks) and treatment (two levels: SRP alone and SRP + laser). †† Statistically significant at p < 0.05. Note: Significant time × treatment interactions were observed for PPD (p = 0.008, partial η² = 0.065) and CAL (p = 0.022, partial η² = 0.054), indicating that the patterns of improvement over time differed significantly between the two treatment groups. Significant main effects of treatment were also observed for PPD (p = 0.004, partial η² = 0.134) and CAL (p = 0.016, partial η² = 0.096). No significant time × treatment interactions were observed for PI (p = 0.702), GI (p = 0.583), or BOP (p = 0.091), indicating no statistically significant evidence of differences between the groups in the patterns of improvement in these parameters.

Parameter	Source	Sum of Squares	df	Mean Square	F-value	Partial η²	P-value
Plaque index (PI)	Time	31.24	3	10.41	182.45	0.757	<0.001
	Treatment	0.09	1	0.09	1.58	0.027	0.214
	Time × treatment	0.08	3	0.03	0.47	0.008	0.702
	Error	10.14	174	0.06	-	-	-
Gingival index (GI)	Time	28.76	3	9.59	156.82	0.726	<0.001
	Treatment	0.12	1	0.12	1.96	0.033	0.167
	Time × treatment	0.12	3	0.04	0.65	0.011	0.583
	Error	10.64	174	0.06	-	-	-
Probing pocket depth (PPD), mm	Time	118.47	3	39.49	124.82	0.676	<0.001
	Treatment	2.84	1	2.84	8.98	0.134	0.004††
	Time × treatment	3.84	3	1.28	4.05	0.065	0.008††
	Error	55.04	174	0.32	-	-	-
Clinical attachment level (CAL), mm	Time	92.36	3	30.79	89.14	0.602	<0.001
	Treatment	2.14	1	2.14	6.19	0.096	0.016††
	Time × treatment	3.42	3	1.14	3.3	0.054	0.022††
	Error	60.08	174	0.35	-	-	-
Bleeding on probing (BOP), %	Time	43,627.85	3	14,542.62	98.74	0.628	<0.001
	Treatment	718.42	1	718.42	4.88	0.078	0.031
	Time × treatment	1,238.47	3	412.82	2.8	0.046	0.091
	Error	25,624.68	174	147.27	-	-	-

Predictors of clinical attachment gain

The multivariable linear regression analysis evaluating factors associated with CAL gain at 24 weeks is presented in Table 5. After adjustment for age, sex, smoking status, diabetes mellitus, baseline PPD, and baseline CAL, adjunctive DL therapy remained independently associated with greater CAL gain (β = 0.235, p = 0.039). Baseline CAL demonstrated the strongest association with treatment response (β = 0.454, p < 0.001), whereas baseline PPD demonstrated a nonsignificant trend toward an association (p = 0.064). Age, sex, smoking status, and diabetes mellitus were not independently associated with CAL gain. The final model explained 37.4% of the variance in CAL improvement (R² = 0.374; adjusted R² = 0.342).

**Table 5:** Multivariable linear regression analysis of factors associated with clinical attachment level (CAL) gain at 24 weeks. Model summary: R² = 0.374; adjusted R² = 0.342; F(7, 52) = 4.426; p < 0.001. CAL: Clinical attachment level; PPD: Probing pocket depth; SRP: Scaling and root planing. Statistical analysis: Multiple linear regression was performed, with eligible variables entered simultaneously according to a prespecified analytical plan. Variables with p < 0.10 in univariable analyses were entered into the multivariable model. The dependent variable was CAL gain at 24 weeks (mm). The variance inflation factor (VIF) was <2.0 for all variables, indicating no evidence of problematic multicollinearity. †† Statistically significant at p < 0.05. Note: The regression model accounted for 37.4% of the variance in CAL gain (R² = 0.374). Treatment group (β = 0.235, p = 0.039) and baseline CAL (β = 0.454, p < 0.001) remained independently associated with CAL gain after adjustment for the other covariates. Baseline PPD was not statistically significant (p = 0.064), although the direction of the association suggested that deeper initial pockets might be associated with greater attachment gain. Age, sex, smoking status, and diabetes mellitus were not statistically significant predictors in this model.

Variable	Unstandardized Coefficient (B)	Standard Error	Standardized Coefficient (β)	t Value	P-value	95% CI for B
Treatment group	0.398	0.188	0.235	2.117	0.039††	0.022 to 0.774
Baseline CAL (mm)	0.346	0.088	0.454	3.932	<0.001††	0.170 to 0.522
Baseline PPD (mm)	0.204	0.108	0.222	1.889	0.064	-0.012 to 0.420
Age (years)	-0.006	0.008	-0.079	-0.75	0.456	-0.022 to 0.010
Sex (male vs. female)	-0.112	0.172	-0.066	-0.651	0.518	-0.456 to 0.232
Smoking status (smoker vs. nonsmoker)	-0.287	0.204	-0.158	-1.407	0.165	-0.695 to 0.121
Diabetes mellitus (yes vs. no)	-0.342	0.256	-0.148	-1.336	0.187	-0.854 to 0.170

Subgroup and sensitivity analyses

To evaluate the robustness of the primary findings, subgroup analyses were performed after stratification by smoking status (smokers vs. nonsmokers). Among nonsmokers (n = 45), the SRP + laser group demonstrated significantly greater CAL gain at 24 weeks than the SRP-alone group (mean difference, 0.44 mm; 95% CI, 0.08-0.80; p = 0.018). Among smokers (n = 15), the between-group difference was not statistically significant (mean difference, 0.29 mm; 95% CI, -0.34 to 0.92; p = 0.342), although the small sample size in this subgroup limits interpretation. These findings indicate that the between-group difference was statistically significant among nonsmokers but not among smokers. However, the small smoker subgroup limits conclusions regarding the effect of smoking on the benefits of adjunctive laser therapy, and further investigation is required.

**Figure 4:**
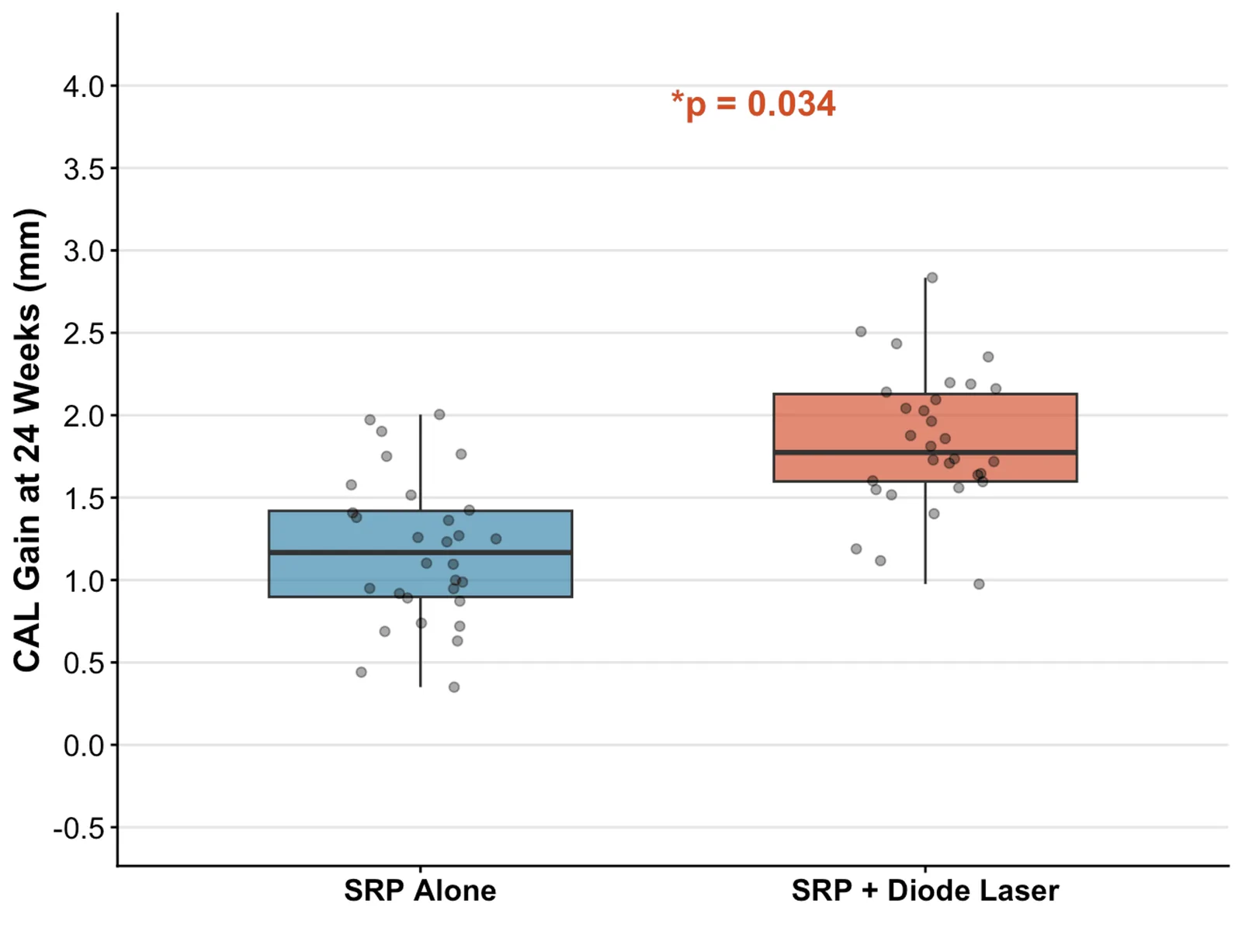
Box-and-whisker plot showing the distribution of CAL gain. Box-and-whisker plot showing the distribution of CAL gain at 24 weeks in the SRP-alone (n = 30) and SRP + diode laser (n = 30) groups. The boxes represent the IQRs, the horizontal lines indicate the medians, and the whiskers extend to the minimum and maximum values. Individual dots represent participant-level observations. The SRP + diode laser group demonstrated significantly greater mean CAL gain than the SRP-alone group (p = 0.034; independent-samples t-test). SRP: Scaling and root planing; CAL: Clinical attachment level.

The distribution of CAL gain at 24 weeks showed greater median improvement and less variability in the SRP + laser group than in the SRP-alone group (Figure 4).

**Figure 5:**
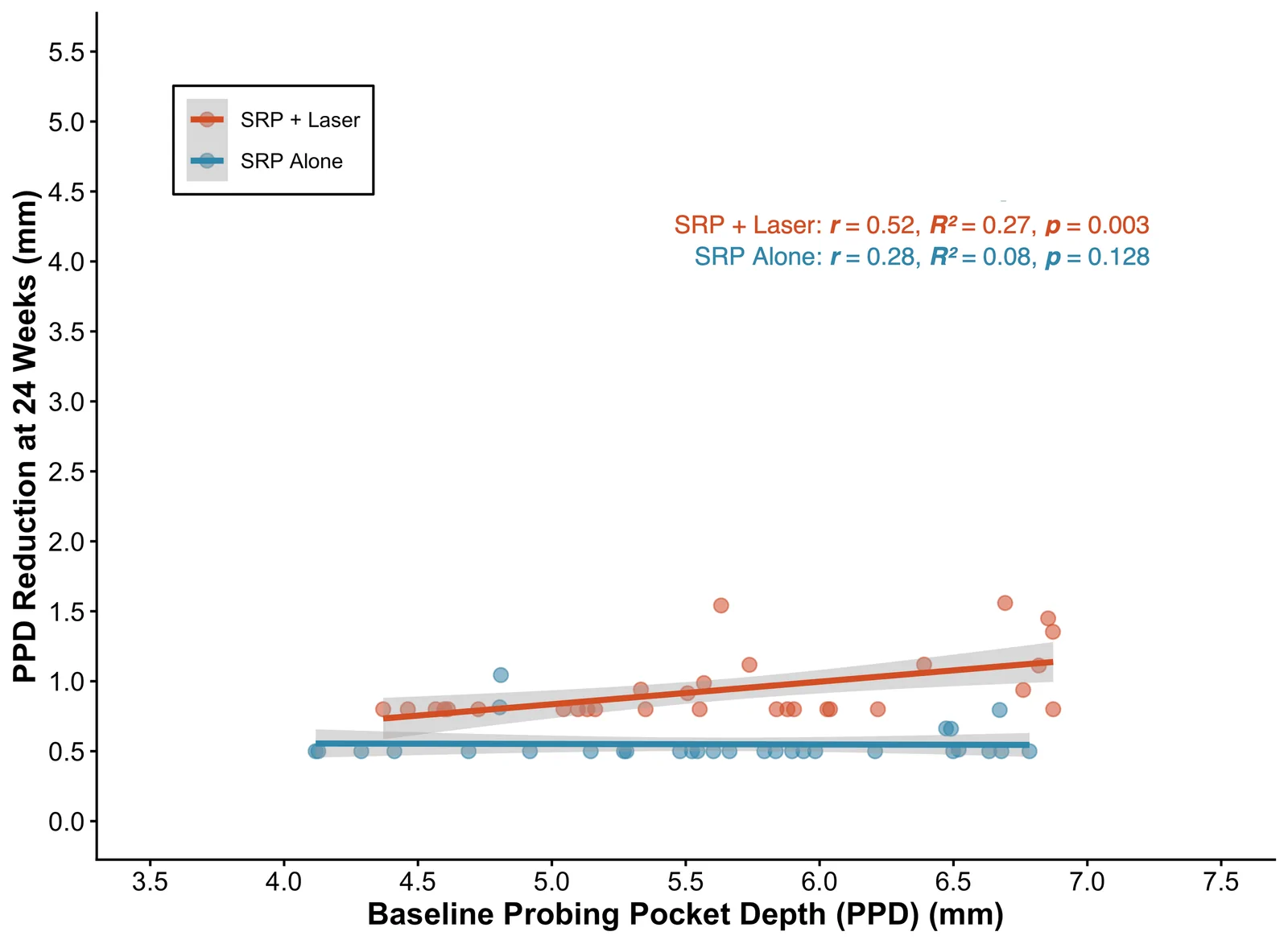
Scatter plot showing the simple linear regression relationship between baseline PPD and PPD reduction. Scatter plot showing the simple linear regression relationship between baseline PPD and PPD reduction at 24 weeks in the SRP-alone (n = 30) and SRP + diode laser (n = 30) groups. Regression lines and shaded 95% confidence bands are shown for each group. The SRP + diode laser group demonstrated a moderate positive correlation (r = 0.52, R² = 0.27, p = 0.003), indicating that participants with deeper baseline pockets experienced greater PPD reductions in the unadjusted analysis. A weaker, nonsignificant correlation was observed in the SRP-alone group (r = 0.28, R² = 0.08, p = 0.128). SRP: Scaling and root planing; PPD: Probing pocket depth.

In an unadjusted simple linear regression analysis, a moderate positive correlation between baseline PPD and PPD reduction was observed in the SRP + laser group (r = 0.52, R² = 0.27, p = 0.003), whereas the correlation was weaker and nonsignificant in the SRP-alone group (r = 0.28, R² = 0.08, p = 0.128) (Figure 5).

Safety and patient-reported outcomes

Safety and patient-reported outcomes are summarized in Table 6. All participants completed the study, and no serious adverse events were reported during the 24-week follow-up period.

**Table 6:** Adverse events and patient-reported outcomes during the 24-week follow-up. VAS: Visual analog scale; SRP: Scaling and root planing; df: Degrees of freedom; d: Cohen’s d; φ: Phi coefficient. For discomfort, VAS scores ranged from 0 (no discomfort) to 10 (worst possible discomfort); for satisfaction, scores ranged from 0 (not satisfied) to 10 (best possible satisfaction). Cramér’s V was interpreted as small = 0.1, medium = 0.3, and large = 0.5. Statistical analysis: Independent-samples t-test for continuous variables and chi-square or Fisher’s exact test for categorical variables, as appropriate. Note: Retention was high, with all participants completing the 24-week follow-up. No serious adverse events were reported. Minor transient sensitivity was the most commonly reported complaint. One participant in the SRP-alone group reported moderate discomfort requiring over-the-counter analgesia beyond the first 72 hours. No participants withdrew because of adverse events. Although the differences were not statistically significant, the SRP + laser group consistently reported lower discomfort scores at all time points, with effect sizes in the small-to-moderate range (d = 0.32-0.39). Overall treatment satisfaction was high in both groups.

Outcome	SRP-Alone Group (n = 30)	SRP + Laser Group (n = 30)	Test Statistic	df	P-value	Effect Size
Postoperative sensitivity, n (%)						
None	18 (60.0%)	20 (66.7%)	χ² = 0.287	2	0.592	Cramér’s V = 0.069
Mild (VAS 1-3)	8 (26.7%)	7 (23.3%)				
Moderate (VAS 4-6)	4 (13.3%)	3 (10.0%)				
Severe (VAS 7-10)	0 (0.0%)	0 (0.0%)				
Gingival recession (mm), mean ± SD	0.42 ± 0.31	0.38 ± 0.28	t = 0.524	58	0.602	d = 0.14
Postoperative discomfort (VAS, 0-10), mean ± SD						
Day 1	3.20 ± 1.82	2.67 ± 1.54	t = 1.221	58	0.227	d = 0.32
Day 3	2.13 ± 1.46	1.60 ± 1.28	t = 1.510	58	0.136	d = 0.39
Day 7	1.07 ± 0.98	0.73 ± 0.82	t = 1.463	58	0.149	d = 0.38
Antibiotic use during follow-up, n (%)						
Yes	2 (6.7%)	1 (3.3%)	χ² = 0.218	1	0.64	φ = 0.060
No	28 (93.3%)	29 (96.7%)				
Missed follow-up visits, n (%)						
4 weeks	0 (0.0%)	0 (0.0%)	-	-	-	-
12 weeks	0 (0.0%)	0 (0.0%)	-	-	-	-
24 weeks	0 (0.0%)	0 (0.0%)	-	-	-	-
Overall satisfaction (VAS, 0-10), mean ± SD	8.40 ± 1.12	8.73 ± 0.98	t = −1.216	58	0.229	d = 0.31

Postoperative sensitivity was the most frequently reported adverse event; however, its incidence did not differ significantly between the treatment groups. Similarly, gingival recession, postoperative discomfort, antibiotic use, and overall treatment satisfaction were comparable between the groups (all p > 0.05). Although participants receiving adjunctive DL therapy consistently reported lower postoperative discomfort scores at all assessment time points, these differences did not reach statistical significance. Overall patient satisfaction remained high in both treatment groups.

## Discussion

The present prospective, nonrandomized comparative clinical study evaluated whether adjunctive 940-nm diode laser therapy provides additional clinical benefits over SRP alone in the nonsurgical management of periodontitis. Both treatment modalities produced significant improvements in periodontal health over the 24-week follow-up period. However, adjunctive diode laser therapy was associated with greater reductions in PPD and greater CAL gain than SRP alone. Repeated-measures analysis demonstrated significant time × treatment interactions, and multivariable regression identified adjunctive diode laser therapy as independently associated with CAL gain after adjustment for baseline clinical characteristics.

The clinical improvements observed in both groups are consistent with the established effectiveness of SRP in disrupting subgingival biofilm and promoting periodontal healing [4,5]. The additional benefits associated with adjunctive diode laser therapy are also consistent with findings from recent systematic reviews and comparative clinical studies [6,8-10]. An umbrella review reported modest improvements in PPD (0.30-0.32 mm) and CAL (0.1-0.35 mm) with adjunctive diode laser therapy at three to six months [16]. Although the magnitude of improvement observed in the present study was modest (0.36 mm PPD reduction and 0.41 mm CAL gain), it is comparable with previously reported outcomes despite differences in laser parameters and study protocols [6-10]. A split-mouth RCT using the same wavelength reported greater CAL and BOP improvements at selected premolar sites in the laser group [17]. Gains of this magnitude have been associated with a reduced risk of disease progression and tooth loss in longitudinal studies, supporting the clinical relevance of the observed effect.

The observed treatment effect is biologically plausible. In addition to reducing the microbial burden, diode laser irradiation has been reported to facilitate de-epithelialization of the periodontal pocket, improve local hemostasis, and promote tissue repair by stimulating fibroblast proliferation, collagen synthesis, and angiogenesis [6-10]. These mechanisms may contribute to enhanced periodontal wound healing and improved attachment gain following conventional mechanical instrumentation.

Although PI, GI, and BOP improved significantly within both groups, no significant between-group differences were observed. As both groups received identical oral hygiene instructions and supportive periodontal maintenance, similar improvements in plaque control and gingival inflammation were expected. These findings suggest that the principal advantage of adjunctive diode laser therapy may lie in enhancing subgingival healing rather than improving supragingival plaque control, which is consistent with its proposed mechanism of action [6,8-10].

Adjunctive diode laser therapy was well tolerated, with no serious adverse events or significant differences in postoperative discomfort, postoperative sensitivity, gingival recession, or antibiotic use between the groups, supporting the short-term tolerability of the treatment protocol used [7-10]. A case report using the 940-nm Biolase Epic X laser also reported substantial improvements and minimal complications, consistent with the findings of the present study [18].

Several limitations should be acknowledged. Treatment allocation was based on participant preference rather than randomization, introducing the possibility of selection bias despite the absence of statistically significant baseline differences, blinded outcome assessment, and multivariable adjustment. The study was conducted at a single center with a relatively modest sample size and a follow-up period of 24 weeks, which may limit the generalizability of the findings. In addition, microbiological, immunological, and follow-up radiographic outcomes were not evaluated, while patient-reported outcomes were limited to postoperative discomfort and treatment satisfaction. Subgroup analyses, particularly among smokers, were also limited by the small sample size. Consequently, these findings should be interpreted as evidence from a prospective comparative effectiveness study rather than a randomized controlled trial.

Future multicenter studies with larger cohorts, longer follow-up periods, standardized laser protocols, and comprehensive clinical, microbiological, biomarker, and patient-reported outcome assessments are warranted to further clarify the long-term effectiveness, biological mechanisms, and cost-effectiveness of adjunctive diode laser therapy in periodontal practice.

## Conclusions

Within the limitations of this prospective nonrandomized comparative clinical study, adjunctive 940-nm diode laser therapy combined with SRP was associated with better clinical outcomes than SRP alone in patients with periodontitis. Although both treatment modalities significantly improved periodontal health over 24 weeks, adjunctive laser therapy resulted in greater PPD reduction and CAL gain while maintaining a favorable safety profile. Improvements in PI, GI, and BOP were comparable between the groups, suggesting that the primary clinical advantage of laser therapy may relate to improvements in PPD and CAL rather than plaque control or gingival inflammation.

These findings suggest that adjunctive diode laser therapy may be a useful complement to conventional nonsurgical periodontal treatment, particularly at sites with moderate-to-deep periodontal pockets. Larger multicenter studies with longer follow-up periods and standardized treatment protocols are warranted to confirm the long-term clinical benefits, biological mechanisms, and cost-effectiveness of adjunctive diode laser therapy in routine periodontal practice.
